# The predictive validity of three self-report screening instruments for identifying frail older people in the community

**DOI:** 10.1186/1471-2458-12-69

**Published:** 2012-01-23

**Authors:** Ramon Daniels, Erik van Rossum, Anna Beurskens, Wim van den Heuvel, Luc de Witte

**Affiliations:** 1 Faculty of Health and Care, Zuyd University of Applied Sciences, PO Box 550, 6400 AN Heerlen, The Netherlands; 2 Centre of Research on Autonomy and Participation, Zuyd University of Applied Sciences, PO Box 550, 6400 AN Heerlen, The Netherlands; 3 Centre of Research on Technology in Care, Zuyd University of Applied Sciences, PO Box 550, 6400 AN Heerlen, The Netherlands; 4 School for Public Health and Primary Care, Maastricht University, PO Box 616, 6200 MD Maastricht, The Netherlands; 5 University Medical Centre Groningen, University of Groningen, PO Box 30, 0019700 RB Groningen, The Netherlands

## Abstract

**Background:**

If brief and easy to use self report screening tools are available to identify frail elderly, this may avoid costs and unnecessary assessment of healthy people. This study investigates the predictive validity of three self-report instruments for identifying community-dwelling frail elderly.

**Methods:**

This is a prospective study with 1-year follow-up among community-dwelling elderly aged 70 or older (n = 430) to test sensitivity, specificity, and positive and negative predicted values of the Groningen Frailty Indicator, Tilburg Frailty Indicator and Sherbrooke Postal Questionnaire on development of disabilities, hospital admission and mortality. Odds ratios were calculated to compare frail versus non-frail groups for their risk for the adverse outcomes.

**Results:**

Adjusted odds ratios show that those identified as frail have more than twice the risk (GFI, 2.62; TFI, 2.00; SPQ, 2,49) for developing disabilities compared to the non-frail group; those identified as frail by the TFI and SPQ have more than twice the risk of being admitted to a hospital. Sensitivity and specificity for development of disabilities are 71% and 63% (GFI), 62% and 71% (TFI) and 83% and 48% (SPQ). Regarding mortality, sensitivity for all tools are about 70% and specificity between 41% and 61%. For hospital admission, SPQ scores the highest for sensitivity (76%).

**Conclusion:**

All three instruments do have potential to identify older persons at risk, but their predictive power is not sufficient yet. Further research on these and other instruments is needed to improve targeting frail elderly.

## Background

The prevalence of frailty increases with age. Frail elderly have a higher risk of disabilities, fall incidents, hospitalization, institutionalization, and death compared to non-frail elderly [1]. With regard to a growing frail population, prevention of adverse outcomes in community-dwelling frail older people is considered to be a priority for research and clinical practice in geriatric care [2].

Effective screening of frailty is crucial in optimizing care to this vulnerable group [3]. Although various tools have been developed, there is not yet a standardized and valid method to screen for frailty. Several authors [4,5] emphasize a two-step approach in preventive interventions, in which screening is followed by extensive assessment. If brief and easy to use self report screening tools are available, this approach may avoid costs and unnecessary assessment of healthy people. It is, therefore, important to study whether tools can predict relevant outcomes associated with frailty. We focused on three available instruments for community-dwelling elderly care: the Groningen Frailty Indicator (GFI) [6], the Tilburg Frailty Indicator (TFI) [7] and the Sherbrooke Postal Questionnaire (SPQ) [5].

All instruments were developed to screen for frailty as a first step in identification. In a Canadian sample of community-dwelling older people, sufficient predictive validity for the SPQ, with regard to functional decline, was reported [5]. There are also positive indications for its predictive value with regard to requirements for further assessment [8], use of emergency services [9], and mortality [10]. We found in a Dutch sample of community-dwelling older people that the GFI and TFI have high internal consistency and construct validity in contrast to the SPQ [11]. In a cross-sectional study, Gobbens et al. [7] reported strong associations between TFI scores and quality of life, disability, and use of nursing and informal care. No longitudinal studies on the predictive values of the GFI and TFI have been conducted yet. The aim of this study is to compare these values of the GFI, TFI and SPQ for relevant adverse outcomes in community-dwelling frail elderly: the development of disabilities, hospital admission, and mortality.

## Methods

### Study design and participants

A longitudinal study was conducted in a sample of 687 community-dwelling older people living in the areas of Limburg and Utrecht in the Netherlands. Older people were identified between November 2008 and April 2009 (T1) from the panels of four general practitioners (GPs). All persons aged 70 years or above from each of the panels received a letter from their GP with an invitation to fill in a questionnaire. The questionnaire included the three frailty instruments (GFI, TFI, SPQ) and an instrument that measures disability with respect to activities of daily living: the Groningen Activity Restriction Scale (GARS) [12]. After 2 weeks, a reminder was sent to non-responders. A pilot study showed that this postal procedure was feasible [13].

One year later (T2), the same questionnaire was sent again to all participants who had provided written consent and responded to the first measurement, except those who had been admitted to a nursing home. This time a question about admission to a hospital in the previous year was added. The study did not require approval from an ethical committee according to Dutch law [14]. Participants gave their written informed consent based on a patient information letter that accompanied the questionnaire. This letter was formulated according to guidelines of good clinical practice. General practitioners were not informed about the frailty states of their patients.

### Data collection

The three frailty instruments and the disability measure are briefly described below. For an overview of all items of the three instruments see Additional file 1: *Frailty Instruments: Overview of all items*.

#### Frailty instruments

The Groningen Frailty Indicator (GFI), developed in the Netherlands by Steverink and colleagues [6], is a screening instrument for determining the level of frailty. It consists of fifteen items, and focuses on the loss of functions and resources in four domains of functioning: physical (nine items), cognitive (one item), social (three items), and psychological (two items). Most items can be answered with 'yes' or 'no'. For the cognitive and psychosocial items, the option 'sometimes' is added. Scores on the GFI range from zero to fifteen. A total score of four or higher is considered as moderately to severely frail [6,15].

The Dutch Tilburg Frailty Indicator (TFI) has recently been described by Gobbens and colleagues [7] and consists of two subscales. The first subscale (ten items) comprises socio-demographic data and data about life-events and chronic diseases. The analyses of predictive values focuses on the second subscale, which determines the level of frailty. This subscale consists of fifteen items about physical (eight items), social (three items), and psychological factors (four items), including one item about cognition. Most items can be answered with 'yes' or 'no'. For the psychological items, the option 'sometimes' is added. Scores for the TFI range from zero to fifteen. A score of five or higher is considered to be associated with frailty [7].

The Sherbrooke Postal Questionnaire (SPQ) was developed in Canada by Hébert and colleagues [5] and consists of six items. These focus on the physical (four items), social (one item), and cognitive (one item) domains of functioning. Items can be answered with 'yes' or 'no'. Scores range from zero to six. Those scoring two or higher, or who do not respond to the questionnaire, are considered to have an increased risk for functional decline and, therefore, are assumed to be frail. It should be noted that in the present study non-responders were excluded from the analyses.

#### Outcome measures

The Groningen Activity and Restriction Scale (GARS) [12] is a valid and reliable instrument for measuring disability. The first subscale is about activities of daily living (ADL) (eleven items). The second subscale relates to instrumental activities of daily living (IADL) (seven items). Items can be answered on a four point scale. In line with the GARS manual [16], the items were dichotomized into being independent or being dependent regarding performance of an activity. Development of disability was defined as (at least) one new disability, meaning a change on at least one of the 18 items of the GARS from being independent to being dependent. Regarding hospital admission, we asked "have you been admitted to a hospital in the previous year". A hospital stay, for at least 1 day, was regarded as hospital admission. The GP provided data at T2 about persons who had died during follow up.

### Statistical analysis

All statistical analyses were performed using SPSS for Windows, version 18.0. In preparing data, missings on the GFI, TFI, and SPQ (if less than 25% of all scale items) on T1 were imputed by means of case mean substitution [17]. To investigate whether baseline characteristics of participants who were lost to follow-up differed from those who remained in the sample, the independent samples t-tests, chi-square tests and Kendall's tau-c tests were used. The areas under the receiver operating characteristic (ROC) curve (AUC) for the proposed cut off points were calculated to compare the accuracy of the instruments. With these points sensitivity, specificity, and positive and negative predicted values were determined for development of disabilities, mortality, and hospital admission. To avoid ceiling effects in calculating predictive values regarding development of disabilities, those scoring 60 or higher on the GARS at T1 (n = 4) were removed from analyses. Odds ratios (OR) were calculated to compare frail versus non-frail groups for their risk for the adverse outcomes. OR's adjusted for age, sex, GARS score on T1, education, and income were calculated using logistic regression (fixed model).

## Results

Of the 687 elderly people who were invited, 532 (77%) returned the baseline postal questionnaire and gave written consent. One year later, the same questionnaire was sent to 514 of these 532 participants (15 participants had died and three had been admitted to a nursing home); 440 (86%) returned the second questionnaire. Ten participants were excluded as there were clear signs that the respondents were not the same at T1 and T2. The frailty instrument with the greatest number of excluded respondents due to missing values (> 25% missing values) was the SPQ (n = 8). For the GFI and the TFI, one and two persons, respectively, were excluded due to missing values. On an item-level the number of missing values ranged from zero to eight (GFI), from zero to twelve (TFI) and from one to ten (SPQ). The average number of missing values per item was 2.4, 5.1 and 5.3 for the GFI, the TFI and the SPQ, respectively.

Finally, 430 participants were included in the analyses (63% of the original sample) for calculating the predictive values for developing disabilities and hospital admissions (for mortality analysis n = 532).

Characteristics of participants at T1 and T2, as well as non-responders at T2, are listed in Table 1. The mean age at T1 was 77.2 years (SD = 5.5) and about 60% were women. Nearly half of the sample (48.6%) had a secondary educational level. A large proportion of people (42.4%) had a net income of more than € 1500 (per month/per household). When using the proposed cut-off points, the GFI detected 245 frail cases (46.3%). The TFI and the SPQ identified 40.2% and 59.1% of the population as frail, respectively. The mean GARS total score at T1 was 24.9 (range 18-72) and 24.2 at T2, indicating hardly any change in disability on a population level over a 1 year period. In comparison with participants that remained in the sample (n = 430), non-responders on T2 (n = 74) had slightly more difficulties in performing activities of daily living, a lower educational background, and lower income. However, these non-responders were not significantly more frail.

**Table 1:** Patient characteristics

	T1 (n = 532)	T2 (n = 430)	Non-responders (n = 74)	**Significance-level difference responders/non-responders at T1 **^**†**^
**Age**				

- 70-74	193 (36.3)	158 (36.7)	24 (32.4)	*p *= 0.46
- 75-79	193 (36.3)	157 (36.5)	28 (37.8)	
- ≥ 80	146 (27.4)	115 (26.7)	22 (29.7)	

Women	311 (58.5)	259 (60.2)	39 (52.7)	*p *= 0.22

Education*				
- None/primary education	186 (35.7)	140 (32.9)	31 (41.9)	*p *= 0.05
- Secondary education	253 (48.6)	213 (50.1)	30 (40.5)	
- Higher education	82 (15.7)	72 (16.9)	8 (10.8)	

Income*				
- ≤900	93 (18.7)	64 (15.6)	24 (32.4)	*p *< 0.001
- 901 - 1500	194 (39.0)	157 (38.4)	28 (37.8)	
- ≥1501	211 (42.4)	188 (46.0)	15 (20.3)	

Frail				
- GFI (≥4)	245 (46.3)	198 (46.0)	32 (43.2)	*p *= 0.63
- TFI (≥5)	211 (40.2)	162 (38.2)	35 (47.9)	*p *= 0.12
- SPQ (≥2)	305 (59.1)	250 (58.1)	37 (52.9)	*p *= 0.28

Disability (mean)				
- GARS total (18-72)	24.9 (sd 9.3)	24.2 (sd 8.3)	28.2 (sd 12.6)	*p *< 0.001
- GARS-ADL (11-44)	13.9 (sd 4.5)	13.5 (sd 3.9)	15.6 (sd 6.5)	*p*< 0.001
- GARS-HDL (7-28)	11.1 (sd 5.4)	10.7 (sd 4.9)	12.7 (sd 6.7)	*p *< 0.001

Mortality	-	15 (2.8%)	-	

Hospital admission	-	75 (17.4%)	-	

*Due to missings small differences between n and numbers of participant reported for education and income can occur

Comparison of these subgroups on baseline scores

Out of 430 older persons, 105 (24%) experienced development of disability; they became dependent on at least one (other) of the 18 GARS activities during follow-up (≥1 new disability). In total 184 new disabilities occurred in 1 year; 35% in ADL and 65% in IADL. Disability in taking care of feet and toenails accounted for 55% of ADL disability, followed by disability in going up and down the stairs (14%). Not being able to do shopping independently accounted for 26% of IADL disability, followed by disability in light household activities and heavy household activities (both 18%). 75 persons (17%) were admitted to a hospital during follow-up. Fifteen persons had died, and three became nursing home residents.

Table 2 shows how the development of disability, mortality and hospital admission are distributed according to the frailty scores. In all cases elderly identified by any of the three instruments as frail had more adverse outcomes in the following year than those in the non-frail group. For example, in the group identified as frail by the GFI, 38% developed new disabilities while this proportion was 13% in the non-frail group.

**Table 2:** Distribution of development of disability, mortality and hospital admission according to frailty scores

	Functional decline		Mortality		Hospital admission	
	(N = 426)*		(N = 532)*		(N = 430)*	
	+	-	+	-	+	-
GFI: +	74 (38%)	120 (62%)	11 (4%)	234 (96%)	39 (20%)	159 (80%)
GFI: -	30 (13%)	200 (87%)	4 (1%)	280 (99%)	36 (16%)	194 (84%)

TFI: +	64 (41%)	93 (59%)	10 (5%)	201 (95%)	39 (24%)	122 (76%)
TFI: -	39 (15%)	224 (85%)	5 (2%)	309 (98%)	35 (13%)	228 (87%)

SPQ: +	85 (34%)	162 (66%)	10 (3%)	295 (97%)	55 (22%)	195 (78%)
SPQ: -	18 (11%)	150 (89%)	4 (2%)	207 (98%)	17 (10%)	152 (90%)

*Due to missings small differences between n and numbers of participant reported for each instrument can occur

Table 3 shows that values for area under curve for all three instruments at the proposed cut off points related to all dependent variables (development of disability, mortality, and hospital admission) are between 0.54 and 0.67. Based on the proposed cut off points, diagnostic values were calculated as shown in Table 3. Compared to 71% for the GFI and 62% for the TFI, the SPQ has the highest sensitivity (83%) regarding development of disabilities. Specificity is lowest for the SPQ (48%). The positive predicted values of the GFI, TFI, and SPQ are up to 40% and all have high (at least 85%) negative predicted values. Regarding mortality, sensitivity for all tools are about 70% and specificity between 41% and 61%. Positive predicted values are very low, and negative predicted values all very high. Regarding hospital admission, SPQ scores the highest for sensitivity (76%), compared to GFI (52%) and TFI (53%). In contrast, specificity is lower for SPQ (44%) in comparison with GFI (55%) and TFI (65%).

**Table 3:** Diagnostic values of the screening tools* for development of disabilities, mortality and hospital admission

	Sensitivity (95% C.I.)	Specificity (95% C.I.)	Positive predicted value (95% C.I.)	Negative predicted value (95% C.I.)	Area under the Curve (95% C.I.)
**Development of Disabilities **(n = 426)					

GFI	71 (62-79)	63 (57-68)	38 (31-45)	87 (81-90)	67 (61-73)
TFI	62 (52-71)	71 (65-76)	40 (33-49)	85 (80-89)	66 (60-72)
SPQ	83 (74-89)	48 (42-54)	34 (29-41)	89 (83-93)	65 (60-71)

**Mortality **(n = 532)					

GFI	73 (44-91)	54 (50-58)	4 (2-8)	98 (96-99)	64 (50-77)
TFI	67 (39-87)	61 (56-65)	5 (2-8)	98 (96-99)	64 (50-78)
SPQ	71 (42-90)	41 (37-46)	3 (1-6)	98 (94-98)	56 (42-71)

**Hospital admission **(n = 430)					

GFI	52 (40-64)	55 (50-60)	20 (15-26)	84 (79-89)	54 (46-61)
TFI	53 (41-64)	65 (60-70)	24 (18-32)	87 (82-90)	60 (52-67)
SPQ	76 (65-85)	44 (39-49)	22 (17-28)	90 (84,94)	60 (53-67)

*Cut-off scores: GFI ≥4, TFI ≥5, SPQ ≥ 2

Odds ratios comparing frail and non-frail groups are presented in Table 4. The unadjusted odds ratios for elderly identified as frail to develop disability are for all three instruments significant and close to 4 (GFI, 4,11; TFI, 3.96;SPQ, 4.36). Adjusted odds ratios for disability are, as expected, lower but still significant for all instruments. The unadjusted odds ratios for mortality are only significant for GFI (3.29) and TFI (3.08), but their impact diminishes after adjusting for baseline characteristics and GARS T1 scores. Regarding hospital admission, only the unadjusted odds ratios of TFI and SPQ reach significance.

**Table 4:** Odds ratios Unadjusted and Adjusted for sex, age, income, education, GARS (T1)

	Development of disabilities		Mortality		Hospital admission	
	**OR unadjusted (95% C.I.)**	**OR Adjusted (95% C.I.)**	**OR Unadjusted (95% C.I.)**	**OR Adjusted (95% C.I.)**	**OR unadjusted (95% C.I.)**	**OR Adjusted (95% C.I.)**

GFI†	4.11 * (2.54-6.65)	2.62* (1.48-4.64)	3.29 * (1.03-10.47)	1.35 (0.32-5.76)	1.40 (0.84-2.33)	1.33 (0.73-2.41)

TFI ^†^	3.96 * (2.48-6.30)	2.00 * (1.18-3.57)	3.08 * (1.04-9.13)	1.05 (0.24-4.60)	2.08* (1.26-3.46)	2.59* (1.36-4.90)

SPQ^†^	4.37* (2.51-7.61)	2.49 * (1.35-4.61)	1.75 (0.54-5.67)	0.92 (0.20-4.33)	2.56* (1.40-4.67)	2.42 * (1.27-4.62)

*Significant at *p*≤ 0,05

^†^Cut-off scores: GFI ≥4, TFI ≥5, SPQ ≥ 2

## Discussion

The aim of this study was to compare the predictive values of three short postal screening instruments for identifying community dwelling frail older persons: the Groningen Frailty Indicator (GFI), the Tilburg Frailty Indicator (TFI) and the Sherbrooke Postal Questionnaire (SPQ).

The associated AUC values, between 0.54 and 0.67, indicate poor performance regarding prediction of any of the dependent variables (development of disability, mortality, and hospital admission). Despite high prevalences of frailty (between 40 and 60%), the positive predicted values of the tools are low. The adjusted odds ratios show that those identified as frail have more than twice the risk (GFI, 2.62; TFI, 2.00; SPQ, 2,49) for developing disabilities within 1 year compared to the non-frail group; those identified as frail by the TFI and SPQ have more than twice the risk of being admitted to a hospital.

This is the first time that these three instruments are compared in one study for their predictive values. The postal procedure proved to be feasible with high response rates. A limitation of our study can be that, by dichotomizing development of disabilities, we might have missed more subtle changes in performance of activities. However, from a clinical perspective, a change from independent to dependent seems more important. Previous studies into frailty used a similar approach for the development of disabilities [18,19]. One could argue that the follow-up period of 1 year is too short to monitor relevant adverse outcomes. However, in our study 24% of older persons did develop disabilities over a one-year period, and from a GP perspective, 1 year seems a reasonable timeframe for pro-active elderly care. The study may have been biased due to treatments that participants received (or did not receive) during the follow-up period influencing changes in disability. However, general practitioners were unaware of the frailty state of their patients and if care was received then this is the case for both frail and non frail respondents.

It is likely that cognitive impairments in the target population have affected the validity of the self reported data. Persons with severe impairments may have been part of the non-responders, thereby influencing the underestimation of frailty prevalences. Further, responders with cognitive impairments may have provided non-reliable information in returned questionnaires. No data are available though about the cognitive impairments among the target population. Based on the high response and the minor changes in disability in the population over a 1 year period, we assume that the influence of (severe) cognitive impairments on the validity of the data is small.

Finally, the SPQ was not used according to protocol [5], as non-responders were excluded from analyses. If we had considered non-responders also at risk, this would have resulted at T1 in a frailty prevalence estimate of 67.0% instead of 59.1%.

The prevalence estimates of 40% to 60% found in the present study are high compared to other studies [19,20]. It is important to realize that prevalence estimates strongly depend on the interpretation of the concept of frailty and the approach that is chosen to measure it. The instruments chosen for this study are based on a multifactorial approach to frailty; lower prevalence estimates are found for instruments based on the definition of physical frailty. Interesting is that frailty scores did not change dramatically over a one-year time period. There are several possible explanations. There may have been a balance in the number of older persons with new incidents of frailty and those who were frail and passed away. Further, we have to consider that frailty is a dynamic process including transitions from frail to non-frail. On the other hand, the frailty instruments may not be sensitive enough to detect small changes in frailty status.

Our diagnostic values of the SPQ for development of disabilities are comparable with those Hébert et al. [5] found among elderly persons over 74 (sensitivity 75% and specificity 52%). Gobbens et al. [7] presented for the TFI a sensitivity of 84% and specificity of 76% for identifying frail elderly at risk for disability. However, this was based on a cross-sectional study design. As mentioned earlier, the adjusted odds ratios show that those identified as frail by the GFI, TFI, and SPQ have, more than twice the risk for developing disabilities within 1 year. Sarkisian et al. [21] found in a cohort study that elders identified as frail with the CHS frailty index, as proposed by Fried et al. [20], had a age-adjusted odds ratio of 4.4 for disability over a 4 year period. Ensrud et al. [19] found, in a prospective cohort study for women (≥69) identified frail with the CHS frailty index, a higher age adjusted risk (OR 2.2-2.8) for disability (≥1 new IADL disability) over a period of 4 and a half years. Differences in estimated risks between those and our study may be attributed for a large part to variation in follow-up periods.

There is a public health need for effective interventions targeting community-dwelling frail elderly promoting their independent functioning in daily life [22]. Prevention of disability in frail older persons contributes to the maintenance of quality of life and reduced health care costs [23]. Supporting primary care to address the needs and health risks of frail elderly is a strategy to control costs as it is expected to prevent institutional care and promote consistency and coordination of individual care [24]. A multifactorial and multidisciplinary approach towards disability prevention in community dwelling frail elderly seems promising [25,26]. For an example of an innovative primary care intervention we refer to a description of our disability-prevention programme [27]. Effective screening is a crucial first step in these programmes to select the appropriate target group. Postal screening questionnaires such as the GFI, TFI and SPQ do have potential to identify older persons at risk.

Our previous study [28] showed that extensive assessment after screening is necessary, as the scalability of the instruments is poor. The current study shows that the predictive power of the instruments is not sufficient yet. The high prevalence of frailty may point to the possibility that a substantial proportion of these elderly is pre-frail. In a two-step approach towards screening, the sensitivity will be the most relevant criterion. In that perspective, the SPQ scores best, followed by the GFI. The SPQ has the highest sensitivity (83%) for development of disabilities; though with a specificity of 48%, a large proportion of older persons that do not develop disabilities are identified. A number of 18 out of 103 elderly who developed disabilities were not identified as frail and thus will not receive an additional assessment. General practitioners who wish to start pro-active elderly care could consider the use of a short postal screening tool in combination with strategies to reduce the number of false positives and false negatives. The additional use of clinical judgment with an instrument as the Clinical Frailty Scale [29] after the preliminary screening might be an option to reduce false positives. This judgment could be based on a recent consultation or a new appointment in which the GP focuses on recent transitions in functioning. Still, more research is necessary to optimize screening in community-dwelling frail elderly.

## Conclusion

The Groningen Frailty Indicator, Tilburg Frailty Indicator and Sherbrooke Postal Questionnaire do have potential to identify older persons at risk, but their predictive power is not sufficient yet. Further research on these and other instruments is needed to improve targeting frail elderly.

## Abbreviations

GFI: Groningen frailty indicator; TFI: Tilburg frailty indicator; SPQ: Sherbrooke postal questionnaire; GARS: Groningen activity restriction scale; GP: General practitioner; ADL: Activities of daily living; IADL: Instrumental activities of daily living

## Competing interests

The authors declare that they have no competing interests.

## Authors' contributions

All authors were involved in the design of the study. RD was responsible for data gathering and data analyses and in writing the manuscript. EvR, AB, WvdH and LdW provided valuable comments during the research process and the process of writing the manuscript. All authors have read and approved the final version of the manuscript.

## Pre-publication history

The pre-publication history for this paper can be accessed here:

http://www.biomedcentral.com/1471-2458/12/69/prepub

## Supplementary Material

Additional file 1 **Overview of frailty instruments**. Click here for file
